# Revisiting the early event of African swine fever virus DNA replication

**DOI:** 10.1128/jvi.00584-25

**Published:** 2025-05-30

**Authors:** Wenlian Weng, Hua Wang, Miaomiao Ye, Dongmei Hu, Jiajun Wu, Yajin Qu, Peng Gao, Yongning Zhang, Lei Zhou, Xinna Ge, Xin Guo, Jun Han, Hanchun Yang

**Affiliations:** 1 State Key Laboratory of Veterinary Public Health and Safety, Key Laboratory of Animal Epidemiology of the Ministry of Agriculture and Rural Affairs, China Agricultural University College of Veterinary Medicine 630101 , Beijing, China; 2 China Animal Disease Control Center 668026, Beijing, China; Lerner Research Institute, Cleveland Clinic, Cleveland, Ohio, USA

**Keywords:** African swine fever virus, viral DNA, replication site, transcription site

## Abstract

As a member of nucleocytoplasmic large DNA viruses, African swine fever virus (ASFV) has been proposed to include an intriguing nuclear stage for DNA replication. In this report, we revisited this early event by using the ASFV type II strain HN09 as a model organism. Our studies began with dissecting the early stages of the ASFV life cycle via analyses by viral growth kinetics, electron microscopy, and quantitative PCR (qPCR). We showed that the single replication cycle lasted about 9 h, with the initial early mRNA transcription taking place around 0.5 h upon virus incubation with host cells, a timing that is much earlier than previously found; the viral DNA replication occurred around 4–5 hours post-infection (hpi), coinciding with the formation of early viral factory (VF), followed by virion morphogenesis at 6–9 hpi. The time-course tracking of viral genomic and newly synthesized DNA via EdU (5-ethynyl-2-deoxyuridine) labeling and DNAscope *in situ* hybridization combined with 3D reconstruction revealed that the viral DNA remained all the time in the cytoplasm. Further transcriptional studies by RNAscope targeting three early mRNAs (*CP204L*, *F334L*, and *H359L*) and EU labeling of nascent viral RNAs revealed similar results regarding their localization. In line with these observations, disruption of the nucleocytoplasmic shuttling with either small interfering RNA (siRNA) or chemical inhibitor did not affect ASFV replication. In contrast, the replication of porcine pseudorabies virus (PRV) was significantly reduced. Thus, we provide strong evidence to suggest that ASFV DNA replication does not involve a nuclear stage. Our findings provide great insight into the replication biology of ASFV.

**IMPORTANCE:**

African swine fever virus (ASFV) represents a devastating threat to the global swine industry. This virus has a large genomic size of 170 to 200 kb with a complex virion structure, but how this virus coordinates transcription/replication cascades has remained poorly defined. By using modern techniques, including EdU (5-ethynyl-2-deoxyuridine) and EU labeling, DNAscope and RNAscope, 3D reconstruction, and RNA interference (RNAi), we provide compelling evidence to show that the ASFV life cycle does not involve a nuclear stage, with both viral transcription and DNA replication confined to the cell cytoplasm. Our findings provide important insight into ASFV replication biology and into seeking targets for antiviral drug development.

## INTRODUCTION

African swine fever (ASF) is a highly contagious infectious disease of domestic pigs with a mortality rate of up to 100% and is currently the most serious challenge to the sustainable development of global pork production (1, 2). The first case of ASF was described in 1921 in Kenya (3), and it then spread out of Africa on three occasions to Europe and finally reached the Asia continent in 2018 (4–8), leading to devastating economic losses. As of today, many countries in Europe and Asia have maintained the endemic status with ASF (1, 2, 7, 8). Unfortunately, for the past 100 years, there have been no effective vaccines or antiviral drugs available against this economically troubling disease. The causative agent is African swine fever virus (ASFV), a large double-stranded DNA virus and the sole member of the family *Asfarviridae* (4, 9, 10). Phylogenetically, ASFV belongs to the clade of nucleocytoplasmic large DNA viruses that include poxviruses, iridoviruses, phycodnaviruses, mimiviruses, etc. (10–13). Within this group, ASFV forms a distinct lineage along with poxviruses, and in particular, they share certain common properties, such as genome structure (e.g., hairpin loops and terminal inverted repeats), a set of core genes, and potentially replication strategies (9, 10, 14–16).

ASFV exhibits a particular tropism for the monocyte/macrophage lineage and can enter the macrophages through different pathways, including actin-driven macropinocytosis, clathrin-mediated endocytosis, and apoptotic body-mediated efferocytosis (17–21). Regardless of the entry routes, the viral particles have to traffic to late endosomes, a compartment where the membrane fusion takes place, and the low pH likely also promotes the uncoating process (17, 22, 23). Upon entry, the nucleoid layer of the particle is released (22), likely accompanied by a cascade of early gene transcription, and the virus initiates the assembly of viral factories in the cytoplasm via a so-far-yet-known mechanism. Although most giant viruses replicate in the cytoplasm, ASFV DNA replication was reported about 20 years ago to have a brief, intriguing nuclear stage, in which the nucleus was radio-labeled positive for DNA replication signals (16, 24). This observation is quite surprising considering that ASFV encodes a complete set of DNA replication/transcription system (nowadays we know it through genomic sequencing) and packages a complex array of enzymes into viral particles (25–27). In addition, a short period of nuclear DNA synthesis and then transport out of the nuclear pore does not appear to be a cost-efficient strategy.

In this report, we revisited the early events of the ASFV life cycle. By using EdU (5-ethynyl-2-deoxyuridine) labeling, DNAscope, and RNAscope in combination with confocal microscopy, we investigated the ASFV transcription/replication cascade and provided several lines of evidence showing that ASFV does not have a nuclear stage for genomic DNA replication in primary macrophages. In contrast, the viral DNA and RNA were present all the time in the cell cytoplasm. The studies of viral replication kinetics revealed that the mRNA transcription of the ASFV type II strain HN09 takes place rapidly and could be discerned within half an hour following virus interaction with host cells, a timing that is much earlier than previously thought. The viral DNA replication occurs around 4 hours post-infection (hpi), followed by virion morphogenesis around 6 hpi. The detailed results are described below.

## RESULTS

### Replication dynamics of ASFV in infected PAMs

**Fig 1:**
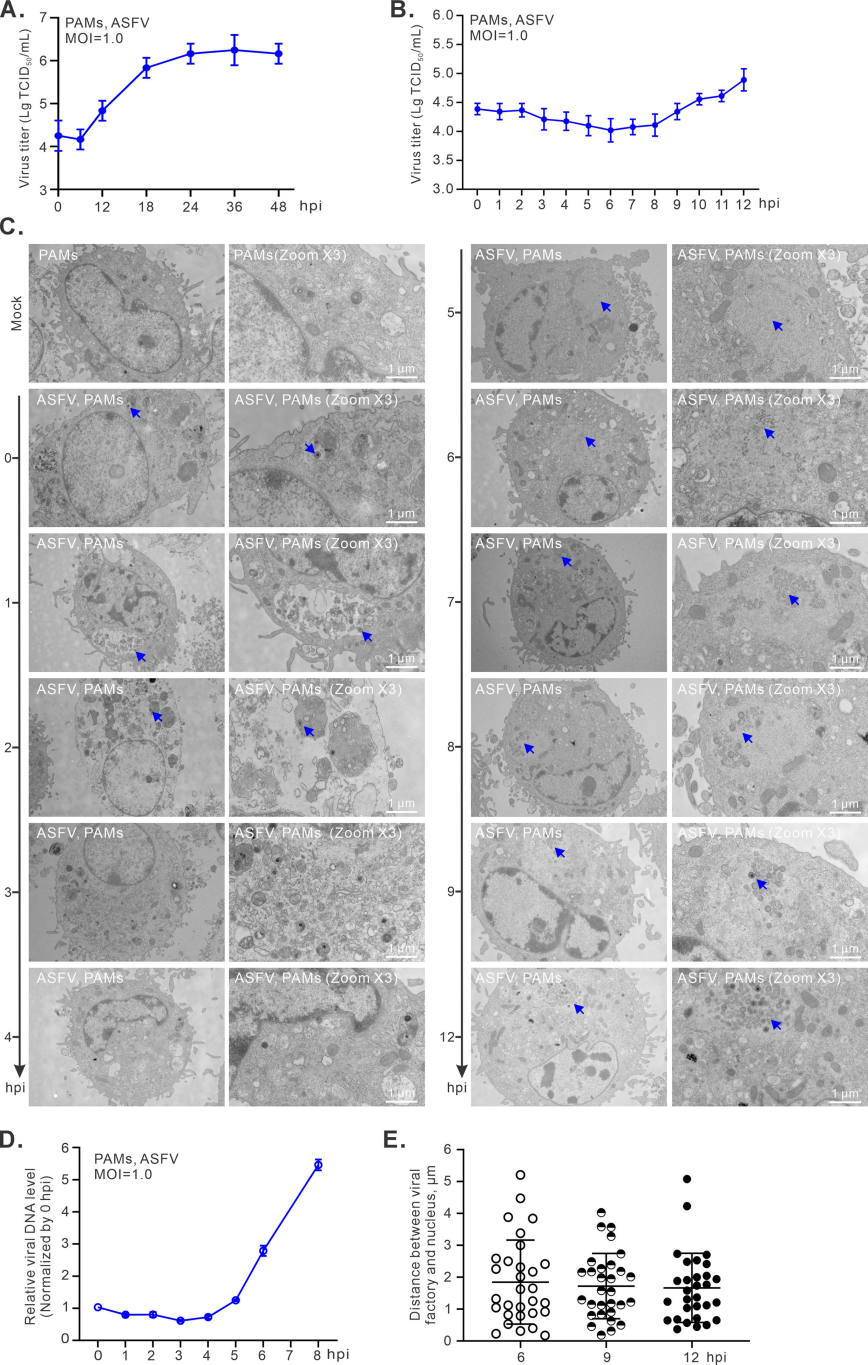
Dissection of the replication dynamics of ASFV in infected PAMs. (**A and B**) Analyses of viral growth kinetics. PAMs were infected with the ASFV strain HN09 at an MOI of 1.0 at 37°C for 1 h. The whole culture was collected at indicated time points post-infection and titrated by endpoint dilution assay. (**C**) TEM analysis of viral replication and morphogenesis. Mock- and ASFV-infected PAMs were fixed at indicated time points and analyzed by TEM. (**D**) Quantitative analysis of the relative viral DNA abundance via qPCR with primers targeting the ASFV gene *F334L* that was normalized against β-actin and then compared to the 0 hpi. Each point represents the mean and standard deviation of three independent experiments. (**E**) Measurement of the distance between the viral factory and the nucleus at the indicated time points. For each time point, 30 infected cells were randomly selected for analysis.

We used the genotype II virulent strain HN09 as a model organism to understand the early events of the ASFV replication cycle. This virus grew well in the primary porcine alveolar macrophages (PAMs) and could reach the replication plateau around 24–36 hpi at an MOI of 1.0 as determined by endpoint dilution assay (TCID_50_) (Fig. 1A). To get further insight into the early kinetics, we performed a more detailed time-course assay. The whole cell culture was collected at 1 h intervals until 12 hpi, a time point that showed the dramatic increase of virion production (Fig. 1A), and we found that the virus titer decreased gradually until 6 hpi and then started to increase around 9 hpi (Fig. 1B). Consistent with virus growth kinetics, the input virions could be observed within the first 2 h following incubation with the host cells as analyzed by transmission electron microscopy (TEM), and they were localized in the endosome-like compartments and then disappeared at 3–5 hpi (Fig. 1C). The earliest visible viral factories (VF) could be discerned at 4–5 hpi; the little crescent stuff that represents the initial assemblies of virions emerged around 6 hpi; the mature virion particles could be largely seen around 9–12 hpi (Fig. 1C). This observation is also in line with the virus growth kinetics (Fig. 1B). Coinciding with the establishment of VF, viral DNA synthesis began around 4 hpi, and a clear accumulation of viral DNA was evident at 5 hpi as analyzed by quantitative PCR (qPCR) with primers targeting ASFV gene *F334L*, an early gene that encodes pF334L, a small subunit of ribonucleotide reductase (28) (Fig. 1D). Thus, the duration of a single replicative cycle defined as a period from virus entry to the emergence of the first mature virion is about 9 h for ASFV strain HN09, and the distinct stages, such as virus entry, viral factory formation/DNA replication (4–5 hpi), and virion assembly (6 hpi), can be distinguished in a sequential, time-dependent manner. Importantly, this dissection enables us in the next step to investigate the early event within the time range of a single viral replicative cycle.

We also measured the positional relationship between VF and nucleus, and the statistical distance between VF and nucleus under TEM typically varied from 0.5 to 2.7 µm with an average distance of 1.6 µm at 6–12 hpi (Fig. 1C and E), suggesting that VF is not necessarily close to the nucleus.

### Revealing the input and replicating DNA in the cytoplasm by EdU labeling

**Fig 2:**
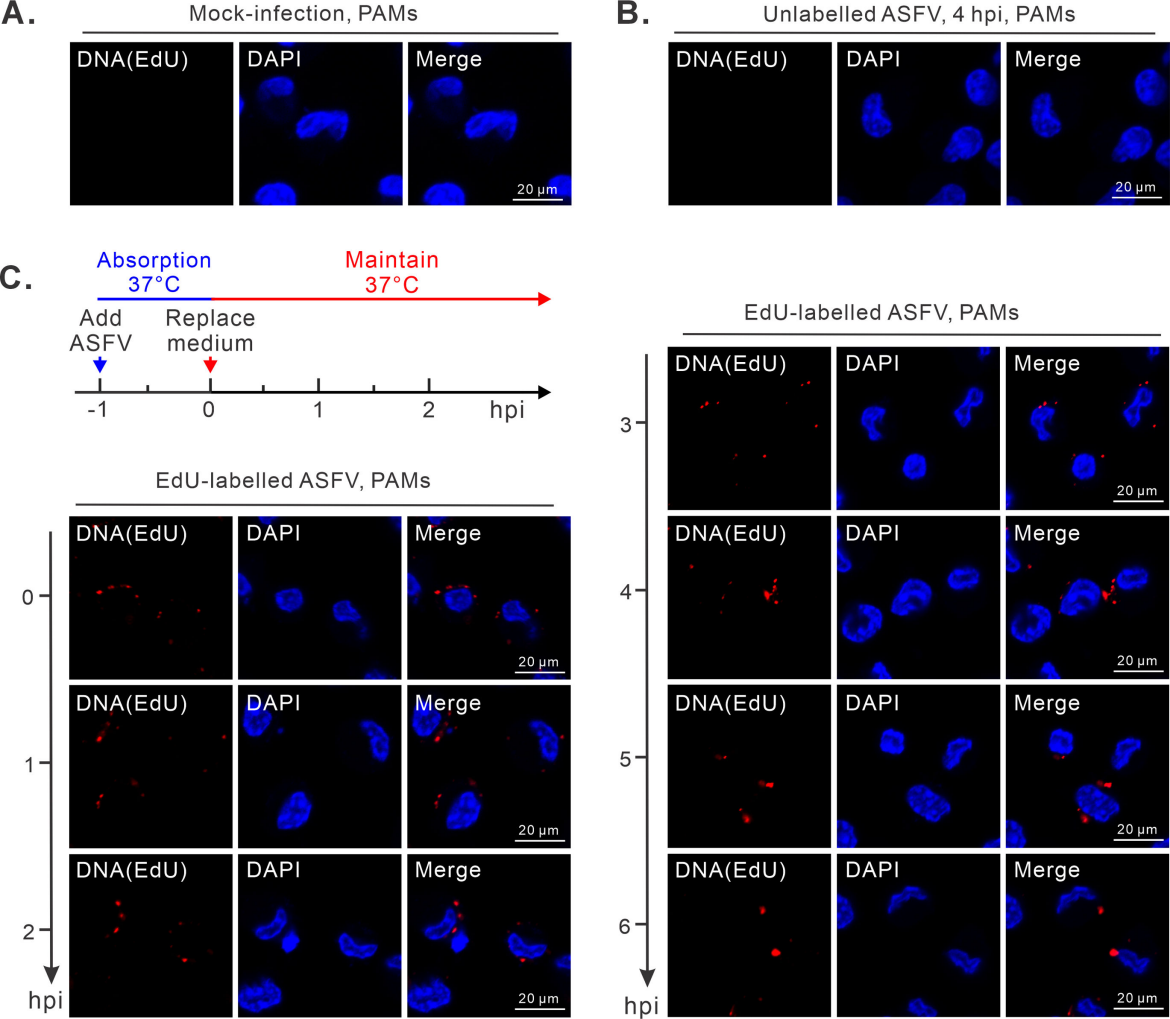
The input ASFV genomic DNAs remain distributed in the cytoplasm. PAMs grown on coverslips in 24-well plates were infected with EdU-labeled or unlabeled ASFV at an MOI of 2.0. After absorption at 37°C for 1 h, the cells were fixed and processed for click chemistry detection of EdU-labeled ASFV DNA at the indicated time points. (**A**) Mock-infected PAMs. (**B**) Unlabeled ASFV-infected PAMs. (**C**) Time-course analysis of EdU-labeled ASFV in infected PAMs.

We next reasoned that if ASFV has a brief nuclear stage for DNA replication, this event should take place before 6 hpi, a time point by which the virion morphogenesis has already taken place (Fig. 1C). We first looked into the trafficking dynamics of ASFV genomic DNA by EdU labeling. The ASFV strain HN09 was propagated in wild swine lung (WSL-R4) cells in the presence of EdU, a nucleoside analog, for 36 h to obtain the progeny virus with genomic DNA labeled by EdU. PAMs were then infected with EdU-labeled ASFV or wild type (WT). At different time points of post-infection, the cells were fixed and subjected to confocal microscopy analysis. As expected, EdU signals were not detectable in mock- or WT-infected PAMs (Fig. 2A and B). In the EdU-labeled ASFV infection group, the signals (viral genomic DNA) were exclusively localized in the cytoplasm of PAMs throughout the time course (0–6 hpi) (Fig. 2C; Fig. S1).

**Fig 3:**
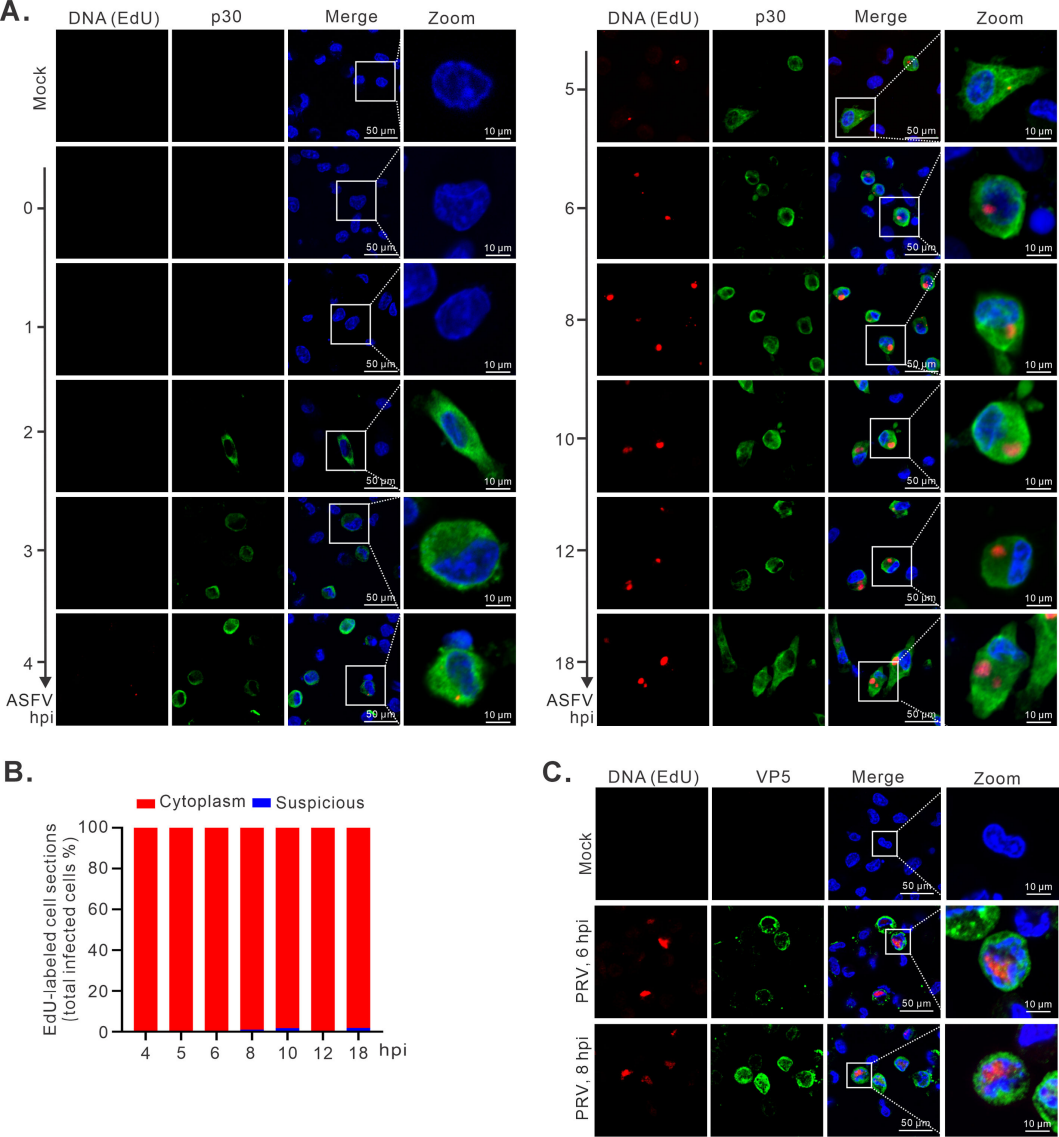
The nascent ASFV DNA is present within the cytoplasm of PAMs. (**A**) Confocal analysis of the subcellular localization of nascent ASFV DNA via EdU-labeling assay. PAMs seeded on coverslips in 24-well plates were infected with ASFV strain HN09 at an MOI of 1.0. The cells were labeled with 25 µM EdU for 30 min prior to fixation at indicated time points, and then detected by click chemistry with EdU and antibodies to viral protein p30. Mock-infected PAMs were used as controls. (**B**) Statistical analysis of the subcellular localization of EdU signals in p30-positive cells. For each time point, 100 infected cells were randomly selected for analysis. (**C**) Confocal analysis of the subcellular localization of nascent PRV DNA via EdU-labeling assay. The same as (**A**), except that PAMs were infected with PRV (MOI = 0.1) and then detected by click chemistry with EdU and antibodies to viral protein VP5.

In the second approach, we tried to label the replicating viral DNA. To this end, PAMs were infected with the ASFV strain HN09 at an MOI of 1.0 on coverslips in 24-well plates and labeled with EdU for 0.5 h for each time point. The cells were then fixed and stained with specific antibodies to p30, a viral early protein that is necessary for ASFV replication (29). Consistent with the replication cascade, the p30 protein was detectable around 2 hpi and lasted throughout the viral life cycle, while the EdU-positive signals (replicating DNA) could not be observed until 4 hpi (Fig. 3A), a time point that coincided with the VF establishment (Fig. 1C). The positive signals for both p30 and EdU could be detected in the same cells from 4 hpi (Fig. 3A; Fig. S2). In addition, the EdU-positive signals exhibited a punctated pattern in the cytoplasm characteristic of VF, and we did not observe such a pattern in the nucleus (Fig. 3A; Fig. S2). In contrast, the EdU-positive signals were observed in the nuclei of pseudorabies virus (PRV)-infected PAMs (Fig. 3C). Further statistical analysis showed that EdU signals in ASFV-infected cells were predominantly localized in the cytoplasm (>98%), while some suspicious perinuclear EdU signals were in close proximity to the cellular nuclear membrane in less than 2% of the ASFV-infected cells after 6 hpi (Fig. 3A and B). This is consistent with the above observation, some VFs, but not most, were close to the nucleus.

### Revelation of viral DNAs in cytoplasm by *in situ* hybridization and 3D reconstruction

**Fig 4:**
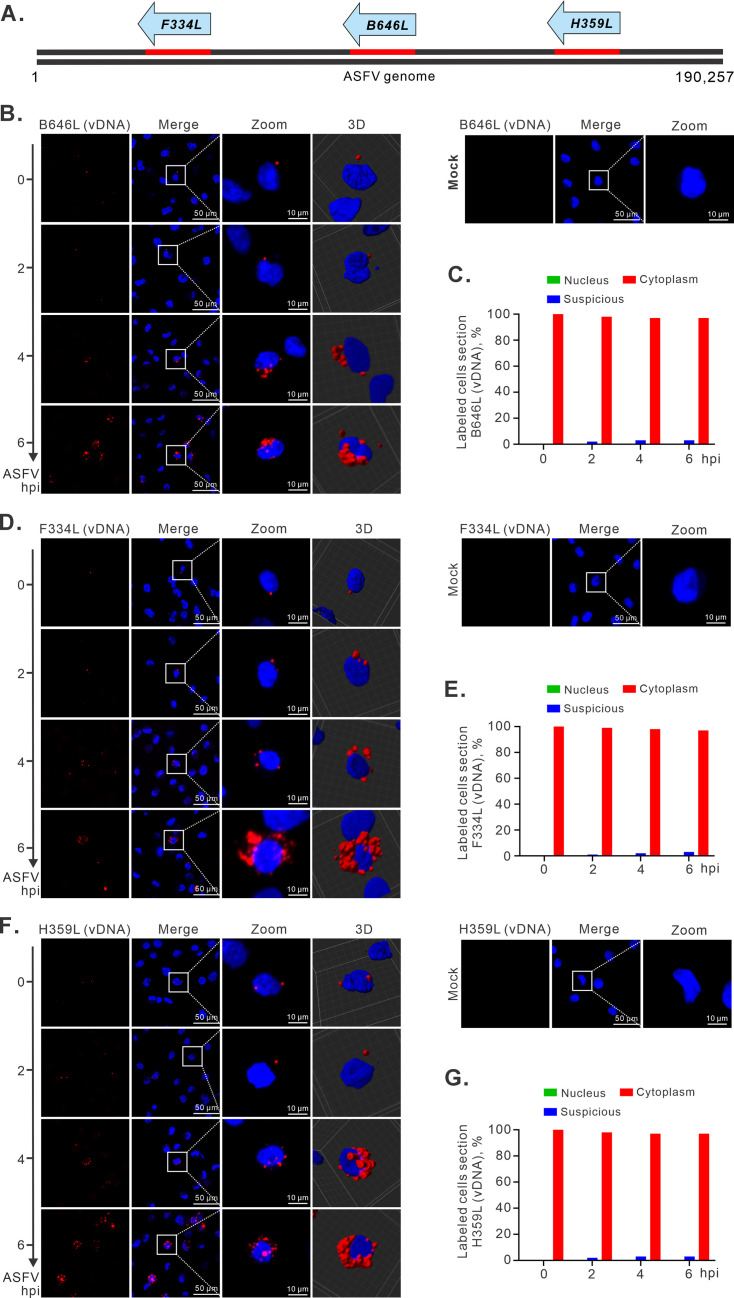
ASFV DNA is localized within the cytoplasm of PAMs. (**A**) Schematic diagram of three DNAscope probes targeting different regions of ASFV antisense DNA. (**B**, **D**, and **F**) Confocal and 3D analysis of the subcellular localization of total ASFV DNA via DNAscope assay. PAMs seeded on coverslips in 24-well plates were infected with ASFV strain HN09 at an MOI of 1.0. The cells were subjected to RNAscope assay with three probes targeting viral gene *B646L* (**B**), *F334L* (**D**), and *H359L* (**F**) at the indicated time points. (**C**, **E**, and **G**) Statistical analysis of the subcellular localization of ASFV DNA. For each time point, 100 infected cells were randomly selected for analysis. The images of C, E, and G corresponded to the *B646L* (vDNA), *F334L* (vDNA), and *H359L* (vDNA). The images were acquired by a Nikon A1 confocal microscope. 3D reconstruction was carried out using Imaris.

One limitation of EdU labeling is that it does not differentiate viral DNA from host DNA. We tried to overcome this problem via DNAscope *in situ* hybridization technology by designing viral-specific probes. Previous studies showed that ASFV may synthesize the small DNA fragments of viral genomic DNA in the nucleus at an early phase, while larger fragments were synthesized in the cytoplasm at a later phase (16). In light of this possibility, we designed three probes targeting different regions (*F334L*, *B646L*, and *H359L*) against their negative strand of ASFV genome DNA (Fig. 4A). The gene *B646L* encodes the major capsid protein p72 (30), and the gene *H359L* encodes pH359L, a subunit of RNA polymerase (31). It is unlikely that only a part of the viral genome DNA goes into the nucleus during replication, and as the viral genome exists as a whole, the positive hybridization signals for any of these three regions will represent the presence of the viral genome in a specific location. Hence, we expect that, if ASFV DNA does have a nuclear stage, the signals for these regions should be positive in the nucleus. It turned out that this is not the case. The hybridization signals for the three regions were all localized within the cytoplasm throughout the time course, and the physical location relationship between signals and nucleus was further confirmed by 3D reconstruction (Fig. 4B through G). In very few cells (1%–3%), there were some signals showing close contact of genomic DNA and host nuclear membrane, which is likely due to a close positioning of viral VF to nucleus and the local curvature of the nuclear membrane, and these were further confirmed by the 3D reconstruction analysis (Fig. 4B, D, and F). To further validate this observation, we conducted the *in situ* hybridization assay in combination with immunofluorescence staining using antibodies to Lamin A/C to indicate the nuclear membrane, and the results showed that ASFV DNA hybridization signals were outside, but not within, the nuclear membrane (Fig. S3). Overall, the above data collectively demonstrate that ASFV DNA does not enter the host cell nucleus, and it replicates in a diffusive manner.

### ASFV RNAs are distributed in the cytoplasm

**Fig 5:**
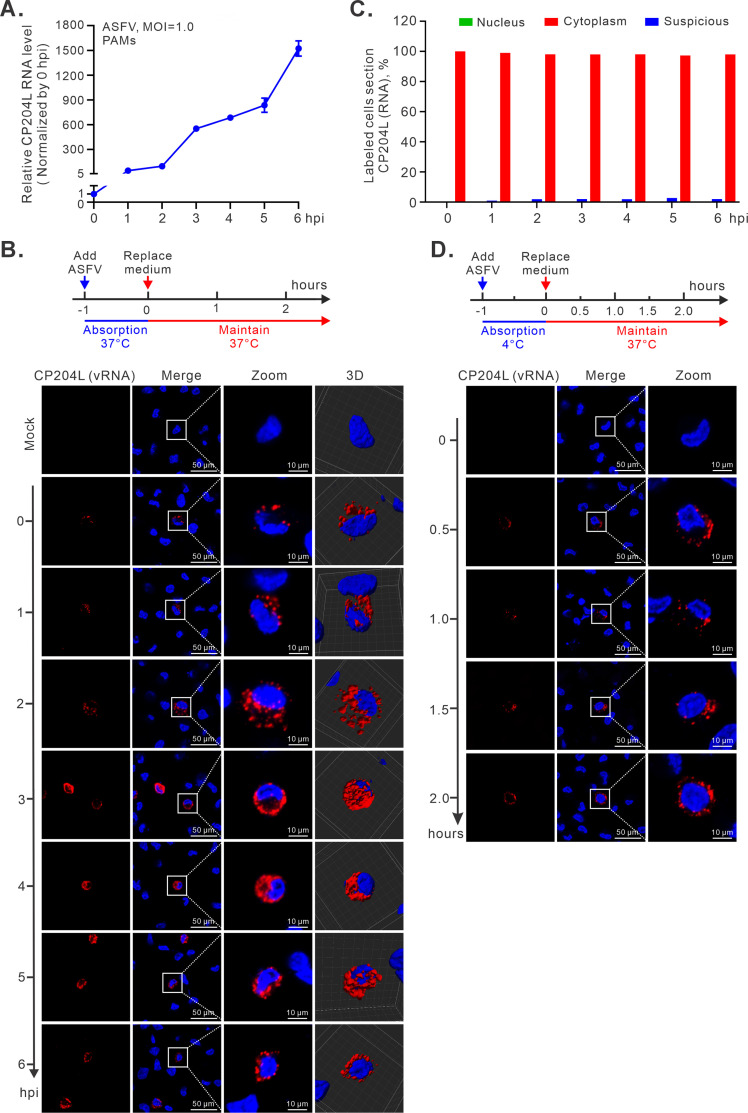
Subcellular distribution of *CP204L* mRNA in the infected PAMs. (**A**) Quantitative analysis of the relative mRNA abundance of ASFV *CP204L* via qPCR that was normalized against β-actin and then compared to the 0 hpi. PAMs were infected with the ASFV strain HN09 at an MOI of 1.0. After absorption for 1 h at 37°C, the total RNAs were extracted at the indicated time points and then determined by RT-qPCR. (**B**) Confocal and 3D analysis of the subcellular localization of ASFV RNA via RNAscope assay. The same as (**A)**, except that ASFV-infected PAMs were fixed for RNAscope with the probe targeting viral *CP204L*. (**C**) Statistical analysis of the subcellular localization of *CP204L* RNA. For each time point, 100 infected cells were randomly selected for analysis. (**D**) Transcription kinetics analysis of ASFV *CP204L* RNA via RNAscope assay. PAMs were infected with ASFV at an MOI of 1.0. After incubation at 4°C for 1 h, the cells were maintained in RPMI-1640 media with 2% FBS at 37°C. It is important to note that we defined ASFV incubation for 1 h at 4°C as 0 hpi in this assay. The images were acquired by the Nikon A1 confocal microscope. 3D reconstruction was carried out using Imaris.

We next investigated the distribution pattern of newly synthesized RNAs in the early stage of the replication cycle. Before DNA replication can take place, some early mRNAs have to be produced. The gene *CP204L* is a good fit as it is an early gene that encodes the p30 protein necessary for viral replication (29). The above study showed that the protein expression of p30 was detectable as early as 2 hpi (Fig. 3A). Moreover, the measurement by quantitative real-time PCR (RT-qPCR) revealed a steady increase of the *CP204L* mRNA in the first 6 h (Fig. 5A), suggesting that the transcription of this gene is a continuous process at least in the early hours of ASFV. Thus, we expect that, if ASFV DNA does go into the nucleus at some stages, this early mRNA should also appear in the nuclear compartment. Unfortunately, this was not the case. Throughout the time course study, the hybridization signals for *CP204L* mRNA remained exclusively in the cytoplasm (Fig. 5B and C). Quite strikingly, the mRNA signals for *CP204L* were detectable in some cells at 0 hpi (Fig. 5B, top panel) (we define virus incubation for 1 h at 37°C as 0 hpi in this assay), and this became more evident at 1 hpi at 37°C (Fig. 5B). As the infection progressed, the signals became even stronger (Fig. 5B). Like the DNA signals, the *CP204L* mRNA (vRNA) displayed a discrete puncta pattern in the cytoplasm of ASFV-infected PAMs (Fig. 5B). As a mock infection control, no signal was detected (Fig. 5B, top panel), suggesting the specificity of the probe. To further dissect the timing for *CP204L* mRNA transcription, the virions were incubated with PAMs at 4°C for 1 h to allow absorption to occur (Fig. 5D, top panel). Afterward, the temperature was raised to 37°C to allow the virus entry. Quite rapidly, the p30 mRNA was detectable at 30 min after endocytosis (Fig. 5D). This result suggests that *CP204L* is an immediate early gene.

To further validate the results, we designed two additional probes targeting viral mRNA of early gene *F334L* and *H359L* (Fig. S4A). The hybridization signals of *F334L* and *H359L* RNA (vRNA) appeared around 1 hpi and also showed a discrete punctate pattern in the cytoplasm (Fig. S4B and C). Again, the statistical analysis of the distribution pattern showed that >97% signals of infected cells were confined to the cytoplasm during the period analyzed, while some signals (<3%) defined as suspicious were localized at the interface between cytoplasm and nucleus (Fig. 5C; Fig. S4D and E). In rare cases, we observe a little signal in the nucleus while most signals remain in the cytoplasm in the same cell, likely due to error diffusion to the nucleus or disruption of the nuclear pore. The accumulation of *F334L* and *H359L* mRNAs was also validated by RT-qPCR (Fig. S4F).

**Fig 6:**
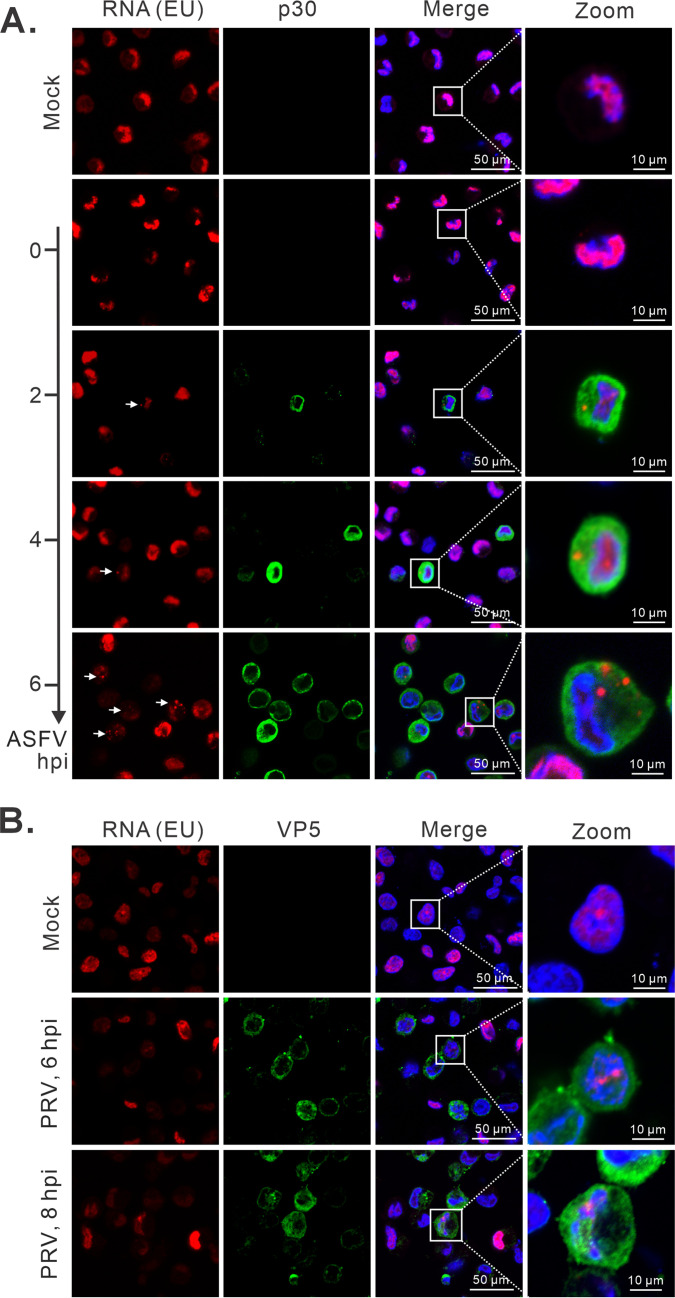
Newly transcribed ASFV RNAs are present within the cytoplasm. PAMs seeded on coverslips in 24-well plates were infected with ASFV strain HN09 (MOI = 1.0) or PRV strain HB1201 (MOI = 0.1). The cells were then exposed to a final concentration of 1 mM EU for 30 min before fixation at indicated time points, and the newly synthesized RNA was detected by EU-click chemistry assay. The viral protein p30 or VP5 was utilized as an indicator of ASFV or PRV infection. (**A**) Newly synthesized RNAs in ASFV-infected PAMs. (**B**) Newly synthesized RNAs in PRV-infected PAMs.

To gain further insights into the distribution pattern of newly synthesized RNAs, mock- and infected-PAMs were incubated with EU (5-ethynyl uridine), an uracil nucleoside analog, for 30 min at the indicated time points before collecting samples for analysis. As expected, in mock-infected cells, the EU signals were mostly in the nucleus, suggesting that there is active transcription going on in the PAMs (Fig. 6A). Interestingly, the EU signals in the nucleus showed a steady decrease in the cells positive for p30 protein staining (Fig. 6A), whereas the signals in the cytoplasm arose around 2 hpi with a distribution pattern in the form of discrete puncta (Fig. 6A), suggesting active transcription in the cytoplasm. For PRV RNA, we did not observe a similar phenomenon in PRV-infected cells (Fig. 6B). These results are consistent with the localization of viral RNAs and suggest that viral RNA transcription occurs in the cytoplasm and also suggest that during infection, ASFV likely shuts down the host mRNA transcription.

### Interference with the nuclear transport function does not affect ASFV replication and DNA localization

**Fig 7:**
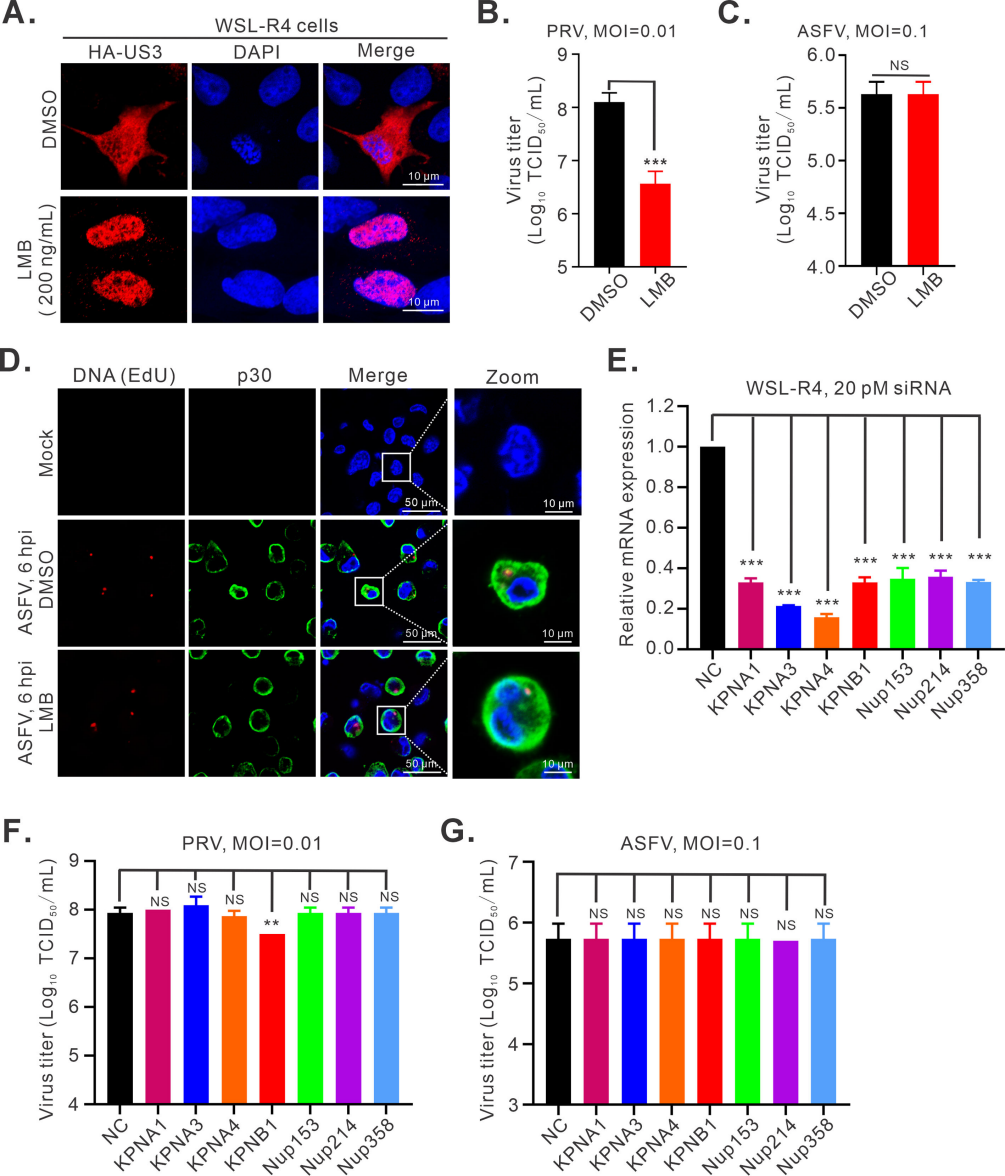
Disruption of nuclear transport function does not affect ASFV DNA replication. (**A**) Effect of LMB treatment on the subcellular localization of PRV US3 protein in transfected cells. (**B**) Effect of LMB treatment on PRV replication. WSL-R4 cells were infected with PRV strain HB1201 at an MOI of 0.01 and maintained in RPMI-1640 media supplemented with LMB for 18 h. (**C**) Effect of LMB treatment on ASFV replication. The same as B, except that the cells were infected with ASFV at an MOI of 0.1 and subjected to virus titer at 36 hpi. (**D**) Confocal analysis of the effect of LMB treatment on nascent ASFV DNA localization. The same as Fig. 3A, except that PAMs were maintained in RPMI-1640 media supplemented with LMB for 6 h. (**E**) Analysis of the RNA interference (RNAi) knockdown efficiency of indicated genes. WSL-R4 cells were transfected with the indicated small interfering RNA (siRNA) (20 pM), and the knockdown efficiencies were assessed by qPCR at 36 h post-transfection with the indicated primers. The relative mRNA level of different genes was normalized against β-actin and then compared to the siNC control. (**F**) Knockdown effect of different genes on PRV replication. At 36 h post-transfection with indicated siRNAs, WSL-R4 cells were infected with PRV strain HB1201 at an MOI of 0.01. At 18 hpi, the whole culture was harvested for viral titration. (**G**) Knockdown effect of different genes on ASFV replication. The same as F, except that the cells were infected with ASFV. Statistical analysis was employed by a two-tailed Student’s t test, and error bars indicate means ± SDs. Asterisks (*) indicate the statistical significance: **P < 0.01; ***P < 0.001; NS, no significance.

Importins and nuclear pore complex (NPC)-associated proteins are specifically required for the nucleocytoplasmic shuttling of intracellular materials and frequently utilized for analysis of the nuclear import/export of viral genomes (32–34). If the ASFV genome does have a nuclear stage, a viral DNA shuttling between the nucleus and cytoplasm is necessary to fulfill this function, and its disruption will have a negative effect on ASFV replication. We tested the hypothesis via two approaches. We first tested the effect of inhibition of nuclear export on viral replication and DNA localization. Leptomycin B (LMB), an inhibitor of exportin-dependent nuclear export of proteins (35), was employed to study its effect on viral growth. As expected, the LMB treatment inhibited the nucleoplasmic shuttling in transfected WSL-R4 cells of PRV protein US3 (Fig. 7A), a protein that has been shown to undergo nucleoplasmic shuttling in transfected cells (36). In untreated cells, US3 was mainly localized in the cytoplasm, whereas treatment with LMB led to significant accumulation of US3 in the nucleus (Fig. 7A). Subsequently, we investigated the effect on ASFV and PRV. The result showed that treatment with LMB led to a significant reduction of PRV replication in WSL-R4 cells (Fig. 7B), but there was no apparent effect on ASFV (Fig. 7C). Accordingly, the cytoplasmic accumulation of *CP204L* mRNA appeared normal (Fig. S5A). In addition, we did not observe any nuclear retention in the LMB-treated cells, a result that is consistent with EU labeling (Fig. 6A; Fig. S5A). The same was true for nascent and total viral DNA accumulation in the cytoplasm (Fig. 7D; Fig. S5B).

In a second approach, we employed the small interfering RNAs (siRNAs) to knock down the crucial importins (also named KPNA and KPNB) and NPC-associated proteins in WSL-R4 cells. The interference efficiency of the siRNA was examined by RT-qPCR, and the mRNA levels of the indicated genes were significantly reduced (Fig. 7E). Unfortunately, none of the RNA interference (RNAi) knockdowns had an effect on ASFV replication (Fig. 7G); but RNAi silencing of importin-β (also named KPNB) selectively inhibited the replication of PRV (Fig. 7F), a DNA virus that is known to replicate in the nucleus (37). Taken together, the above results suggest that nuclear-cytoplasmic shuttling does not affect viral DNA localization and replication compartment.

## DISCUSSION

ASFV stands as the foremost causative agent of pig mortality in the swine industry (38). As a large DNA virus with a complex virion structure, the replication biology of this virus has remained poorly understood, limiting further understanding of viral pathogenesis. One of the most arguable topics pertaining to ASFV DNA replication biology is whether it requires a nuclear period. In this study, by using modern techniques (e.g., DNAscope, RNAscope, 3D reconstruction, EdU labeling, RNAi, etc.), we showed that the ASFV DNA replication and transcription take place exclusively in the cell cytoplasm. Moreover, the viral mRNA transcription occurs much earlier than thought, detectable around 0.5 h upon virus incubation with host cells, and the viral DNA replication takes place around 4–5 hpi, followed by virion assembly at 6–9 hpi (Fig. 1 and 5). Furthermore, the distinct stages of the ASFV single life cycle can be distinguished in a timely manner. These findings have important implications for understanding ASFV biology and pathogenesis. The relevant significance and insights are discussed below.

The earlier studies concerning the importance of the cell nucleus were mainly carried out in two approaches (16, 24, 39). The first is cell enucleation by treatment of Vero cells with cytochalasin B (39). Although the treatment led to significant inhibition of ASFV replication, cytochalasin B is known to have a significant impact on the polymerization of microtubules and potentially other molecules in the cytoplasm, or it may exert a direct effect on viral replication machinery (40–42). The second approach is *in situ* hybridization with viral DNA probes labeled with digoxigenin (16, 24). One caveat in these studies is that they were carried out by two-dimensional analysis, and the lack of sophisticated analysis, such as 3D reconstruction, leads to an insufficient definition of the precise location of viral genomic DNA. In addition, ASFV infection is capable of distorting or disrupting the nuclear membrane (43), and this may lead to mislocalization of some of the probe signals. When taking a closer look, lots of those observed signals that were thought to be in the nucleus are actually in close proximity to the distorted or incaved nuclear membranes.

In this study, our proposal for complete cytoplasmic DNA replication is supported by three lines of evidence. The time-course tracking of viral genomic DNA using EdU labeling and DNAscope analysis provides the most direct evidence. The dissection of the duration of a single life cycle of ASFV and the definition of the different stages of early RNA transcription, DNA replication, and virion assembly by viral growth curve, electron microscopy, and RT-qPCR analyses enabled us to select specific time points (first 6 h) to track the physical location of viral DNA (Fig. 1). We showed that ASFV DNA replication coincided with the establishment of VF at 4–5 hpi (Fig. 1C and D). In this situation, the viral DNA was found to remain in the cytoplasm all the time, and these were further confirmed by statistical analysis and 3D reconstruction (Fig. 4; Fig. S3). The second line of evidence comes from tracking the viral RNA synthesis and the associated distribution pattern. The mRNA of *CP204L* encoding p30 was selected as an indicator as our studies suggested that its mRNA is highly abundant and continuously produced to high levels during the replicative cycle of ASFV (Fig. 5). Unfortunately, we did not observe any of the presence of the *CP204L* mRNA in the nucleus (Fig. 5). The same was true for the mRNAs of *F334L* and *H359L* (Fig. S4). We also used the EU to label the overall nascent RNA transcripts. Although the initial labeling was exclusively host RNAs, as the infection progressed, we observed the formation of punctate RNA foci in the cytoplasm, but not in the nucleus (Fig. 6A), indicating active viral RNA synthesis. Meanwhile, the cellular mRNAs decreased dramatically (Fig. 6A), suggesting a shutdown of cellular RNA synthesis by ASFV.

The third line of evidence comes from genetics and pharmacological approaches. Viruses must deliver their genome into the host cell nucleus if they undertake their DNA replication within the nucleus (32, 44–51). Nuclear import of viral molecules larger than 39 nm is a tightly regulated process that occurs through NPC (32). For example, hepatitis B virus capsids bind and cross the NPC in an importin-α and -β dependent manner (44). The capsid then binds directly to Nup153 at the nuclear basket to trigger disassembly of the capsid and nuclear entry of the viral genome (45). The capsid of Herpes simplex virus 1 docks with a distinct orientation to the NPC cytoplasmic filament by binding to Nup358 in an importin-β and Ran-dependent manner, and then the genome is delivered into the nucleus (46, 47). The capsid of adenovirus was disassembled through binding to Nup358 and Nup214, and subsequent delivery of its genome into the nucleus (48–51). Meanwhile, the previous study showed that PRV polymerase enters the nucleus by interacting with importin α3 and importin β1 (37). In line with the above results, knockdown of some importins and NPC-associated proteins had an effect on PRV, but not on ASFV (Fig. 7F and G), suggesting the specificity of the effect. Consistently, treatment of the cells with LMB resulted in a significant reduction of PRV growth titer but exerted no apparent effect on ASFV (Fig. 7B and C). Overall, our findings are also consistent with the recent whole-genome bioinformatics analyses that ASFV encodes a complete set of replication systems. Thus, ASFV adopts a similar strategy to poxviruses to replicate within the cytoplasm (52).

## MATERIALS AND METHODS

### Cells and viruses

The primary PAMs derived from 1-month-old specific-pathogen-free (SPF) pigs and WSL-R4 were maintained in Roswell Park Memorial Institute (RPMI)-1640 medium supplemented with 10% FBS, 100 U/mL penicillin, and 100 µg/mL streptomycin at 37°C with 5% CO_2_. Type II ASFV strain HN09 (GenBank accession no. MZ614662.1) and PRV strain HB1201 (GenBank accession no. KU057086.1) have been described previously (21, 53). All experiments involving live ASFV were performed in the biosafety level 3 Lab at the China Agricultural University (license number 2022-ASFV-002).

### Antibodies and reagents

Mouse anti-p30 monoclonal antibody was produced by our laboratory at China Agricultural University and used as previously described (21). Lamin A/C monoclonal antibody (sc-376248) was purchased from Santa Cruz Biotechnology. Cell-LightTM EdU Apollo 567 reaction (C10310-1) and Cell-Light EU Apollo 567 RNA (Ribobio, C10316-1) were purchased from Ribobio. Alexa Fluor 488-conjugated goat anti-mouse F(ab')2 fragment (A11017), Alexa Fluor 568-conjugated goat anti-rabbit F(ab')2 fragment (A11011), 4’,6-diamidino-2-phenylindole (DAPI) (62248), and Lipofectamine RNAiMAX (13778150) were purchased from Thermo. RNAscope probes and RNAscope Multiplex Fluorescent Detection Reagents v2 Kit (323110) were purchased from ACD. Blood/Cell/Tissue Genomic DNA Extraction kit (DP304-03) was obtained from Tiangen. ChamQ Universal SYBR qPCR Master Mix (Q712-02-AA) was purchased from Vazyme. TRIzol (MD020) was obtained from Magen. TOROBlue All-in-One qRT Mix with dsDNase (RTQ-104) was purchased from Toroivd. Leptomycin B (HY-16909) was purchased from MedChemExpress.

### Transmission electron microscopy

PAMs were seeded in 25 cm^2^ dishes and uninfected or infected with the ASFV strain HN09 at an MOI of 1.0. After 1 h incubation at 37°C, unbound viruses were removed by washing three times with serum-free culture media. Subsequently, the cells were maintained in fresh culture media containing 2% FBS and incubated at 37°C. At indicated time points, PAMs were harvested by centrifugation and fixed with 4% paraformaldehyde and 2% glutaraldehyde. The cell pellet was then encapsulated in low-melting-point agarose, post-fixed in a cacodylate buffer with 1% osmium tetroxide, and stained en bloc with 1% uranyl acetate. After dehydration with acetone, the sample was embedded in epoxy (TAAB 812 resin). Following polymerization, the sections (80 nm) were obtained and stained with uranyl acetate and lead citrate. Imaging was required in a HITACHI HT7700 electron microscope.

### EdU or EU labeling with clickable nucleosides

To incorporate EdU into the viral genome DNA, WSL-R4 cells cultivated in plastic dishes were initially treated with 2.5 µM EdU for a duration of 24 h. Subsequently, these cells were infected with the ASFV strain HN09 at an MOI of 2.0. Following a 1 h incubation period at 37°C, the cells were washed three times with serum-free culture media to remove the unbound viruses and maintained in fresh culture media, supplemented with 2.5 µM EdU and 2% FBS for 36 h to obtain the progeny virus with genomic DNA labeled by EdU. To separate EdU-labeled virus from cell debris, we collected the cellular supernatant and centrifuged it at 2,000 rpm for 10 min. Then, the supernatant was ultracentrifuged at 28,000 rpm for 2 h and suspended in fresh RPMI-1640 medium for use as EdU-labeled ASFV.

To monitor the incoming ASFV genome DNA, PAMs were seeded on glass coverslips in 24-well dishes and infected with EdU-labeled ASFV at an MOI of 2.0, the wild-type ASFV- and mock-infected PAMs served as the controls. At the different time points, the cells were fixed with 3.7% formaldehyde for 10 min and subjected to Cell-Light EdU Apollo567 reaction (Ribobio, C10310-1) according to the manufacturer’s protocol.

For tracking the newly synthesized DNA and RNA of ASFV and PRV. PAMs were grown on glass coverslips in 24-well dishes and infected with ASFV strain HN09 (MOI = 1.0) or PRV strain HB1201 (MOI = 0.1), mock-infected PAMs served as the control. The cells were exposed to a final concentration of 25 µM EdU or 1 mM EU for 30 min prior to being harvested at the indicated period of post-infection time. After washing three times with phosphate-buffered saline (PBS), the EdU-treated or EU-treated cells were immediately fixed with 3.7% formaldehyde for 10 min and subjected to Cell-Light EdU Apollo 567 reaction (Ribobio, C10310-1) or Cell-Light EU Apollo567 RNA reaction (Ribobio, C10316-1) according to the instructions. And then, the cells were stained with mouse anti-p30 (ASFV) monoclonal antibody or anti-VP5 (PRV) polyclonal antibody for 1 h at room temperature, followed by secondary antibodies. Images were obtained using a Nikon A1 confocal microscope and analyzed by Image J or Imaris.

### Indirect immunofluorescence assay

PAMs were seeded in 24-well dishes and infected with ASFV strain HN09 at an MOI of 1.0. At indicated time points, the cells were subjected to an indirect immunofluorescence assay (IFA) as previously described (54). In brief, the cells were fixed with 3.7% paraformaldehyde for 10 min, permeabilized with PBS containing 0.1% Triton X-100 for 10 min, and then blocked with PBS containing 2% BSA-PBS for 30 min. The samples were then incubated with antibodies to viral protein p30 overnight at 4°C. After being washed three times with PBS for 5 min each, the samples were incubated with secondary antibodies for 1 h. Host nuclear or viral DNA was stained with DAPI (Thermo, 62248) for 10 min and then washed with PBS three times for 5 min each. The cells were imaged using a Nikon A1 confocal microscope.

### Virus infection and titration

To determine the growth kinetics of ASFV strain HN09, PAMs were infected with ASFV strain HN09 at an MOI of 1.0. After absorption for 1 h at 37°C, the cells were washed with RPMI-1640 media and overlaid with fresh media containing 2% FBS. At indicated time points, the whole cell culture was harvested and titrated by the endpoint dilution assay in WSL-R4 cells. Virus titers were expressed as 50% tissue culture infective dose per mL (TCID_50_/mL). Each point represents the mean and standard deviation of three independent experiments.

### Quantitative real-time PCR

To quantify the ASFV DNA copies, 12-well plates of PAMs were infected with ASFV strain HN09 (MOI = 1.0). After absorption for 1 h at 37°C, the cells were washed with acid buffer (135 mM NaCl, 10 mM KCl, 40 mM citric acid, pH 3.0) and overlaid with fresh cultural media containing 2% FBS. At indicated time points, PAMs were scraped off the plates and harvested by centrifugation at 5,000 rpm for 5 min. Then, the total DNA was extracted by using the Blood/Cell/Tissue Genomic DNA Extraction Kit (Tiangen, DP304-03) according to the manufacturer’s protocol. The qPCR was performed using ChamQ Universal SYBR qPCR Master Mix (Vazyme, Q712-02-AA) and MyiQ Real-Time PCR System (Bio-Rad). The reaction conditions were as follows: 5 min at 95°C, 40 cycles of 30 s at 95°C, and 30 s at 60°C. A pair of primers targeting *F334L* was synthesized (Table S1) and used for the qPCR. The cellular β-actin was quantified as the internal control to normalize the total DNA amounts.

To quantify the viral RNA copies, the samples were prepared equal to above. The total RNAs from cultured cells were extracted with TRIzol reagent (Magen, MD020) according to the manufacturer's instructions. The cDNA was synthesized by reverse transcription using TOROBlue All-in-One qRT Mix with dsDNase (Toroivd, RTQ-104) according to the manufacturer’s protocol. qPCR was performed to determine the relative mRNA levels of *CP204L*, *F334L,* or *H359L,* and the housekeeping gene β-actin was used as the internal control to normalize the cDNA amount. The primers used for qPCR are listed in Table S1.

### DNAscope or RNAscope *in situ* hybridization

To detect ASFV DNA (vDNA), *in situ* hybridization was employed by using the RNA-scope Multiplex Fluorescent Detection Reagents v2 Kit (ACD, 323110), and the probes targeting the antisense DNA strand of viral gene *B646L*, *F334L*, and *H359L* were designed and purchased from Advanced Cell Diagnostics (ACD, 320850-I). Then, ASFV DNA was detected with the Probe-V-ASFV-MCP-sense-C1 (ACD, 1193281-C1) targeting *B646L*, Probe-V-ASFV-F334L-sense-C1 (ACD, 1330941-C1) targeting *F334L*, or Probe-V-ASFV-H359L-sense-C1 (ACD, 1330951-C1) targeting *H359L* according to the manufacturer’s protocol. In brief, PAMs on glass coverslips in 24-well dishes were infected with ASFV strain HN09 at an MOI of 1.0. After absorption for 1 h at 37°C, the cells were washed with RPMI-1640 media and overlaid with fresh media containing 2% FBS. At indicated time points, the cells were fixed with 3.7% paraformaldehyde and dehydrated and rehydrated with different concentrations of ethanol. After rehydration, the cells were treated with hydrogen peroxide and protease solution (RNAscope III) diluted in PBS (1:15 dilution). The samples were then incubated with the indicated probes for 2 h in the humidified HybEZ oven at 40°C, followed by a cascade of signal amplification and a series of washing procedures. Nuclei were counterstained with DAPI. Hybridization signals were detected by TSA Vivid 570 (ACD, 7526). For the additional viral protein staining, the cells were fixed again with 3.7% paraformaldehyde and then processed following the normal IFA procedure as above.

To detect ASFV RNA (vRNA), the probes V-ASFV-CP204L-C1 (ACD, 1599641-C1) targeting *CP204L*, V-ASFV-F334L-C2 (ACD, 1310351-C2) targeting *F334L*, and V-ASFV-H359L-C3 (ACD, 1311341-C3) targeting *H359L* were designed and synthesized via ACD. The infective cell samples were prepared and performed equal to the above. Images were obtained using a Nikon A1 confocal microscope and analyzed by Image J and Imaris.

### RNA interference assay

For RNA interference, siRNAs were designed to target different coding regions for each targeted gene (importin α1, importin α3, importin α4, importin β1, Nup153, Nup214, and Nup358). The sequences of siRNAs were listed in Table S2. siRNAs were transfected with Lipofectamine RNAiMAX (Thermo, 3778150) according to the manufacturer’s instructions. Knockdown effect was examined by RT-qPCR at 36 h post-transfection. The primers for qPCR are shown in Table S1. The cellular β-actin was used as the reference gene. For the transfection/infection assay, WSL-R4 cells were infected with ASFV strain HN09 (MOI = 0.1) or PRV strain HB1201 (MOI = 0.01) at 36 h post-transfection of siRNAs, and total viruses were collected at 48 hpi (ASFV) and 18 hpi (PRV), respectively. The samples were titrated on WSL-R4 cells using a standard TCID_50_ assay.

### LMB treatment

WSL-R4 cells seeded onto 24-well plates were optionally pre-treated with LMB (200 ng/mL) for 1 h before being transfected with 0.5 µg of pCMV-HA-US3 plasmid per well. The culture media were replaced with fresh media containing LMB at 4 h post-transfection, and the cells were subjected to IFA at 36 h post-transfection. To assess whether LMB treatment affects the replication and RNA subcellular localization of ASFV or PRV, WSL-R4 cells were treated as the way aforementioned and then infected with either ASFV strain HN09 (MOI = 0.1) or PRV strain HB1201 (MOI = 0.01). The infected cells were then cultured in the media with LMB (200 ng/mL) and collected at indicated times for *in situ* hybridization assay or TCID_50_ assay.

### Statistical analysis

Statistical significance was employed by two-tailed unpaired Student’s t tests in GraphPad Prism version 8.0 (La Jolla, CA, USA). Significance symbols are defined as follows: NS, no significance; *P < 0.05; **P < 0.01; ***P < 0.001. Error bars indicate means ± SD.

## Data Availability

All data are presented in the article and supplemental material.

## SUPPLEMENTAL MATERIAL

Fig. S1 jvi.00584-25-s0001.tif The input ASFV genomic DNAs remain distributed in the cytoplasm.

Fig. S2 jvi.00584-25-s0002.tif The nascent ASFV DNA is present within the cytoplasm of PAMs.

Fig. S3 jvi.00584-25-s0003.tif ASFV DNA is localized outside the nuclear membrane.

Fig. S4 jvi.00584-25-s0004.tif ASFV RNA is localized within the cytoplasm throughout the early stage of infection.

Fig. S5 jvi.00584-25-s0005.tif LMB treatment does not affect ASFV DNA and RNA accumulation in the cytoplasm.

Supplemental material jvi.00584-25-s0006.docx Legends for Fig. S1 to S5; Tables S1 and S2.

ASM does not own the copyrights to Supplemental Material that may be linked to, or accessed through, an article. The authors have granted ASM a non-exclusive, world-wide license to publish the Supplemental Material files. Please contact the corresponding author directly for reuse.
