# Supplementary figures and images for: Revisiting the early event of African swine fever virus DNA replication
Source: J Virol. 2025 May 30;99(7):e00584-25. doi: 10.1128/jvi.00584-25 (PMC12282160; doi:10.1128/jvi.00584-25)

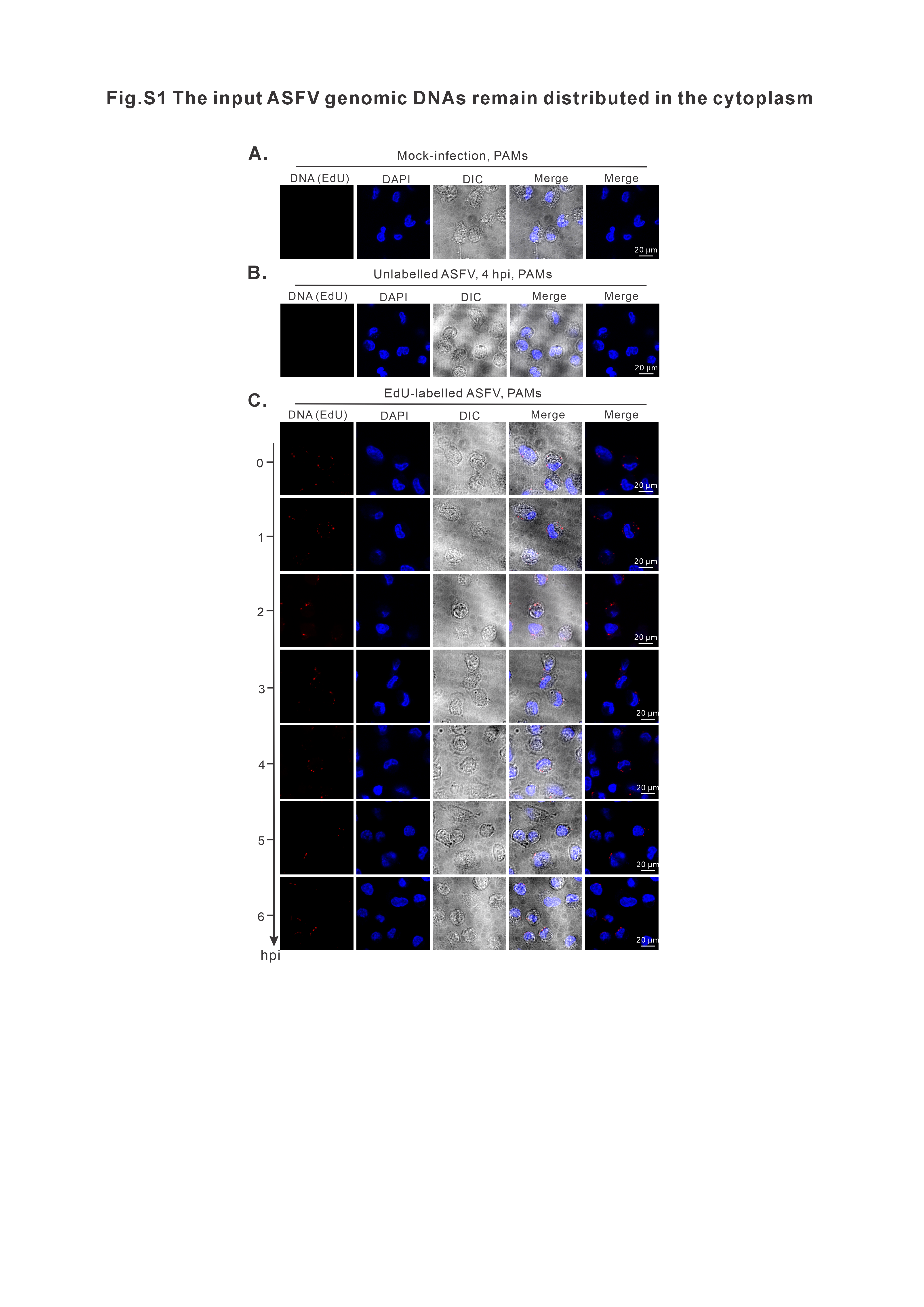

Supplement: Fig. S1 — The input ASFV genomic DNAs remain distributed in the cytoplasm. [file jvi.00584-25-s0001.tif]

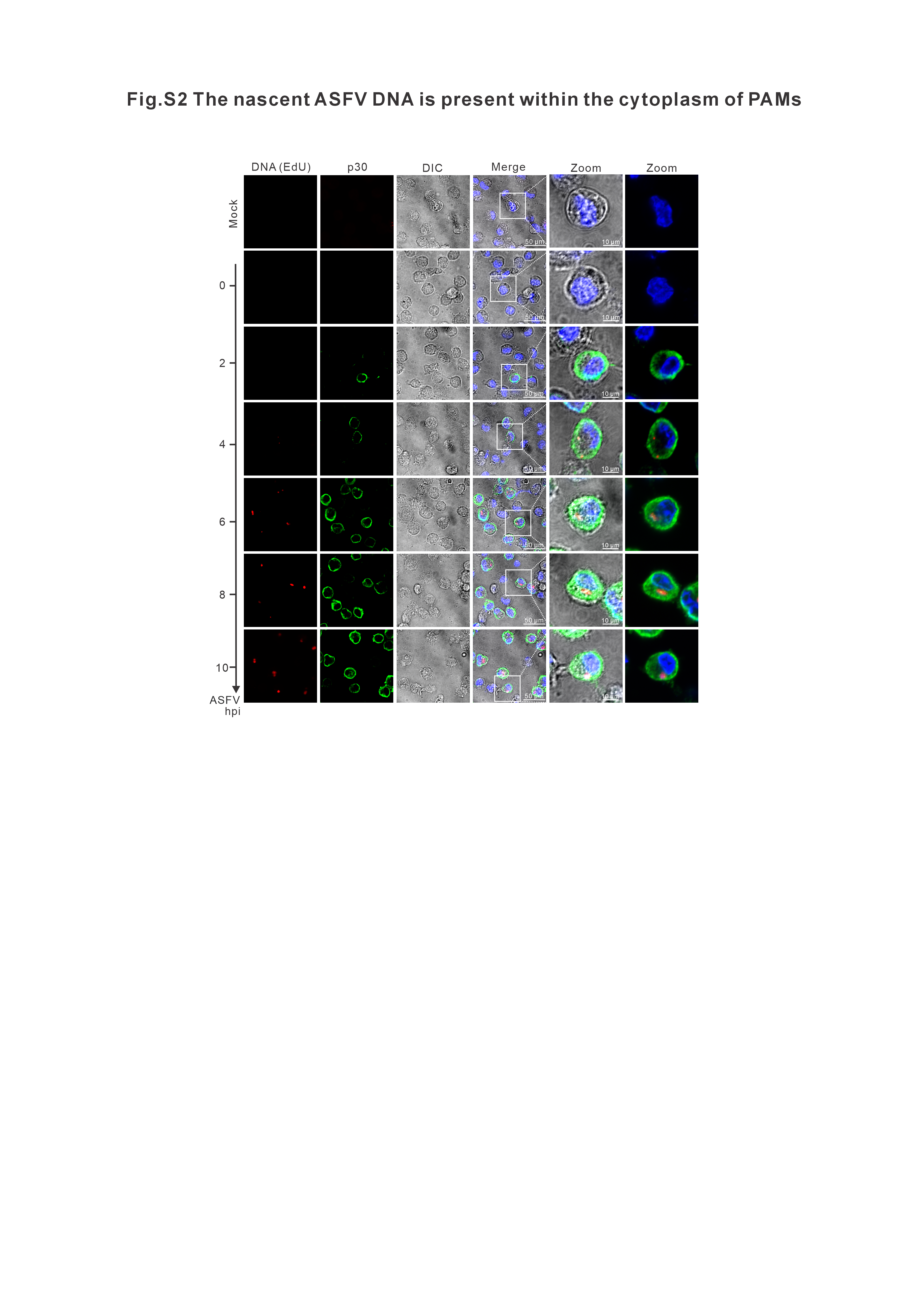

Supplement: Fig. S2 — The nascent ASFV DNA is present within the cytoplasm of PAMs. [file jvi.00584-25-s0002.tif]

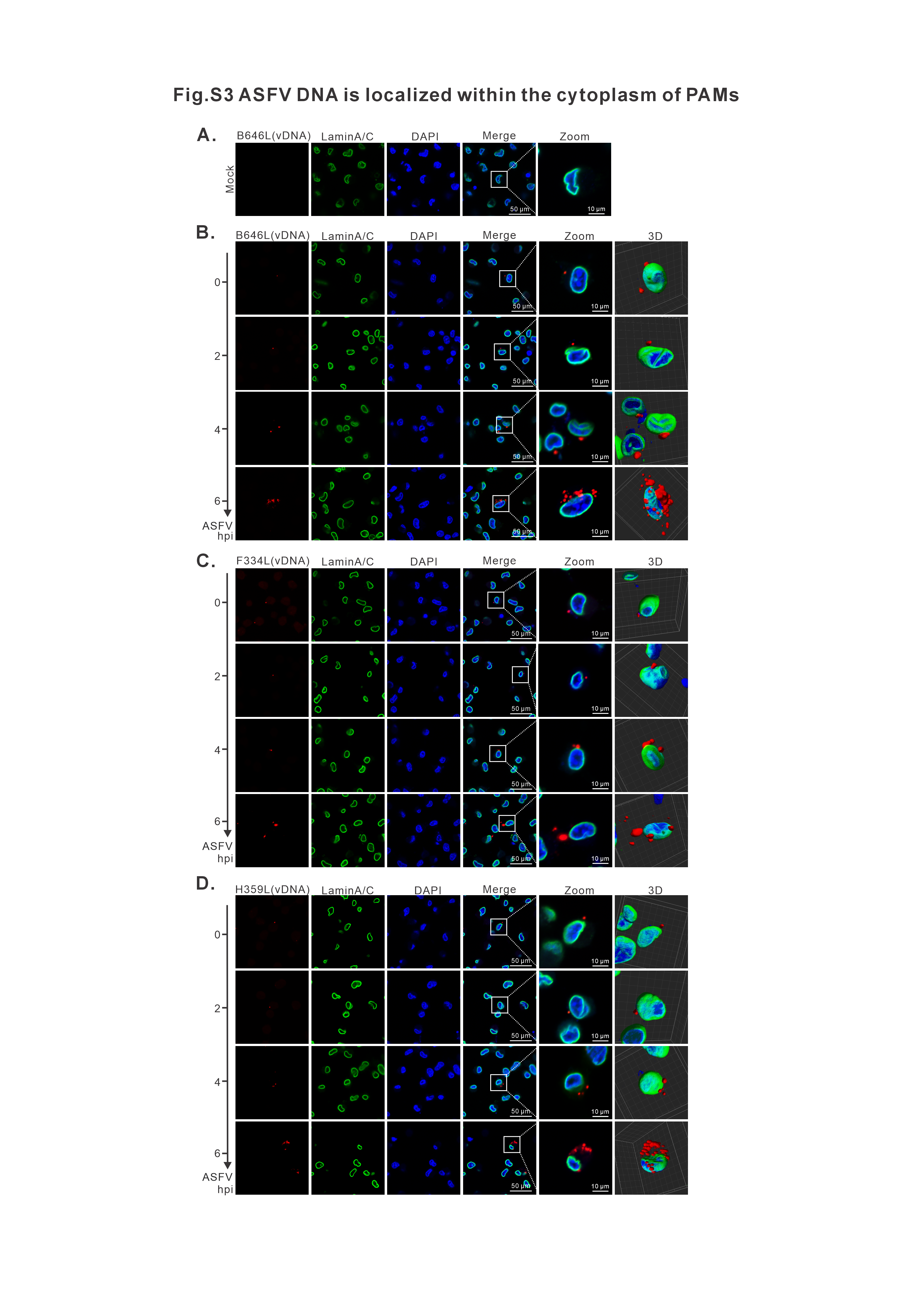

Supplement: Fig. S3 — ASFV DNA is localized outside the nuclear membrane. [file jvi.00584-25-s0003.tif]

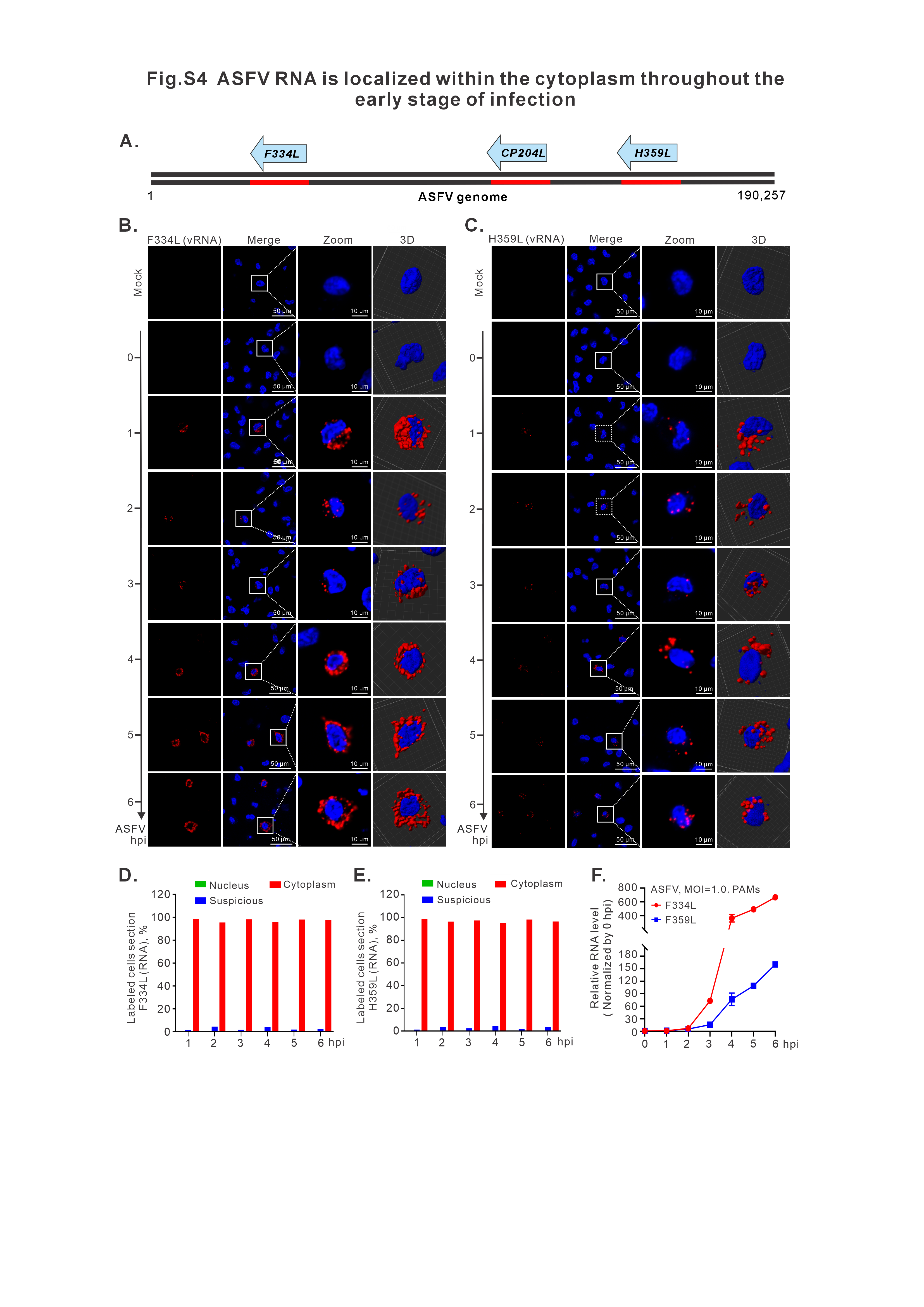

Supplement: Fig. S4 — ASFV RNA is localized within the cytoplasm throughout the early stage of infection. [file jvi.00584-25-s0004.tif]

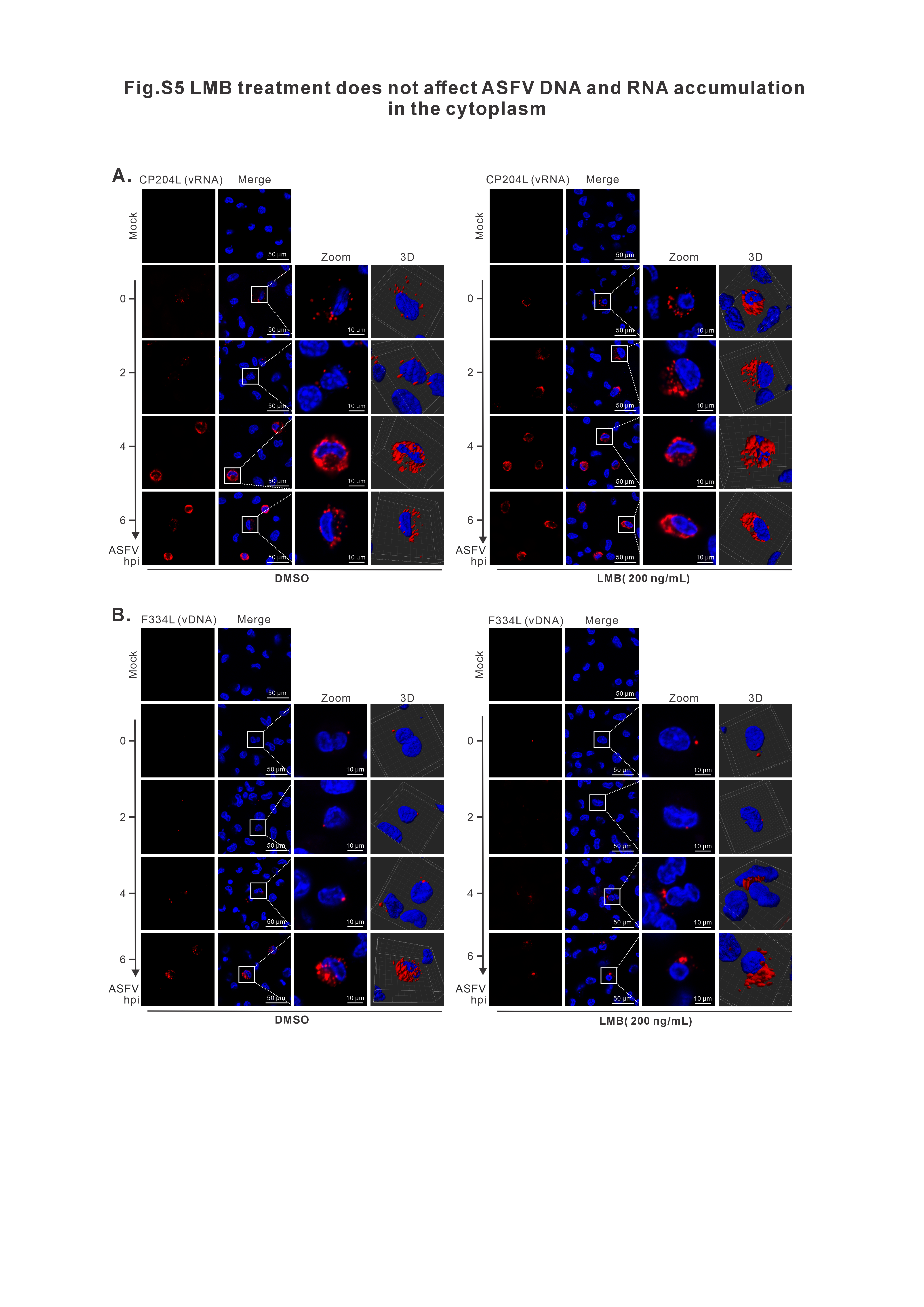

Supplement: Fig. S5 — LMB treatment does not affect ASFV DNA and RNA accumulation in the cytoplasm. [file jvi.00584-25-s0005.tif]
