# Supplementary material for: Revisiting the early event of African swine fever virus DNA replication
Source: J Virol. 2025 May 30;99(7):e00584-25. doi: 10.1128/jvi.00584-25 (PMC12282160; doi:10.1128/jvi.00584-25)
Supplement: Supplemental material — Legends for Fig. S1 to S5; Tables S1 and S2. [file jvi.00584-25-s0006.docx]

**Supplemental Figure legend**

**Fig.S1 The input ASFV genomic DNAs remain distributed in the cytoplasm.** PAMs grown on coverslips in 24-well plates were infected with EdU-labelled or unlabeled ASFV at an MOI of 2.0. After absorption at 37°C for 1 h, the cells were fixed and processed for click chemistry detection of EdU-labeled ASFV DNA at indicated time points. Time-lapse DIC and confocal microscope were employed to image cells. (**A)** Mock infected PAMs. **(B**) Unlabeled ASFV-infected PAMs. (**C**) Time-course analysis of EdU-labelled ASFV in infected PAMs.

**Fig.S2** **The nascent ASFV DNA is present within the cytoplasm of PAMs.** Confocal analysis of the subcellular localization of nascent ASFV DNA via EdU-labeling assay. PAMs seeded on coverslips in 24-well plates were infected with ASFV strain HN09 at an MOI of 1.0. The cells were labeled with 25 μM EdU for 30 min prior to fix at indicated time points, and then detected by click chemistry to EdU and antibodies to viral protein p30. Mock-infected PAMs were used as control. Time-lapse DIC and confocal microscope were employed to image cells. The images were acquired by Nikon A1 confocal microscope.

**Fig.S3 ASFV DNA is localized outside the nuclear membrane.** (**A**) Confocal analysis of the subcellular localization of Lamin A/C in mock-infected PMAs. (**B-D**) Confocal analysis of the subcellular localization of viral DNA in ASFV-infected PMAs. PAMs seeded on coverslips in 24-well plates were infected with ASFV strain HN09 at an MOI of 1.0. The cells were subjected for IFA with the antibody against to Lamin A/C and RNAscope assay with the probes targeting viral gene *B646L* (vDNA) (**B**), *F334L* (vDNA) (**C**), or *H359L* (vDNA) (**D**) at indicated time points. The images were acquired by Nikon A1 confocal microscope. 3D-reconstruction was carried out using Imaris.

**Fig.S4** **ASFV RNA is localized within the cytoplasm throughout the early stage of infection.** (**A**) Schematic diagram of three RNAscope probes targeting three ASFV mRNAs. (**B** and **C**) Confocal and 3D analysis of the subcellular localization of the ASFV RNA. PAMs were infected with ASFV strain HN09 at an MOI of 1.0 and subjected for RNAscope assay with the probes targeting *F334L* (B) and *H359L* (C) at the indicated times. (**D** and **E)** Statistical analysis of the subcellular localization of *F334L* RNA (B) and *H359L* RNA (C). For each time point, 100 infected cells were randomly selected for analysis. (**F**) Quantitative analysis of the relative RNA abundance of ASFV *F334L* and *H359L* via qPCR that was normalized against β-actin and then compared to the 0 hpi. PAMs were infected with ASFV strain HN09 at an MOI of 1.0. After absorption 1 h at 37°C, the total RNAs were extracted and determined by RT-qPCR at indicate times.

**Fig.S5 LMB treatment does not affect ASFV DNA and RNA accumulation in the cytoplasm.** PAMs were infected with ASFV at an MOI of 1.0. After absorption at 37°C for 1 h, the cells were maintained in the RPMI-1640 media with or without LMB (200 ng/mL) and then subjected for RNAscope assay with indicated probes. (**A**) Confocal and 3D analysis of the subcellular localization of ASFV RNA (*CP204L*) in the absence of LMB (left panel) or in the presence of LMB (right panel). (**B**) Confocal and 3D analysis of the subcellular localization of ASFV DNA (*F334L*) in the absence of LMB (left panel) or in the presence of LMB (right panel). The images were acquired by Nikon A1 confocal microscope. 3D-reconstruction was carried out using Imaris.

**Table S1. Primers for qPCR**

| Gene | F (5'-3') | R (5'-3') |
| --- | --- | --- |
| Importin α1-Sus | TATCACAGCTGGAAACAGGGC | GATACAGCCCAATTCTACGAGG |
| Importin α3-Sus | TCAGGGGTTGTCCCATTTCT | AACAACCTGGGTCTGCTCAT |
| Importin α4-Sus | GCAGTTCAGGCTGCTAGGAA | TTTGTCAAAGCCCATGCAGC |
| Importin β1-Sus | AAGCTCGCGGCTACTAATGC | ATCTGGACACTGTGTGGCTT |
| Nup153-Sus | GTATGCAGATGAGGAGGGCG | GCTGACAGTGTAACGCAGGA |
| Nup214-Sus | GAAGTGGAACCCTACCGTCG | GACCACGGTCCCATTCTGTT |
| Nup358-Sus | AAGGCCCGATCTTTCCTTGG | TCCACTGTAAGCCAAGCCAG |
| ASFV-F334L | TGGATCGCCACAAAGTGGTT | GCGGCATTGAAAAACACCCT |
| ASFV-H359L | TTAAGCTTAGGGCCTGCCAC | GCCCGGTGGGTATAAGATCG |
| ASFV-CP204L | CAAGTTGTGTTTCATGCGGG | AGATGCTGAGGATTCCGTCTC |
| β-actin-Sus | CTCCATCATGAAGTGCGACGT | GTGATCTCCTTCTGCATCCTGTC |

F: forward primer; R: reverse primer; Sus: *Sus scrofa*.

**Table S2. Sequences for RNA interference**

| siRNA-Name | sense (5'-3') | antisense (5'-3') |
| --- | --- | --- |
| si-Importin α1 | GCUUGGGUACUGACAAAUATT | UAUUUGUCAGUACCCAAGCTT |
| si-Importin α3 | GUCACACUAGAAGCUAUAUTT | AUAUAGCUUCUAGUGUGACTT |
| si-Importin α4 | GGGCAUUGGGAAAUAUCAUTT | AUGAUAUUUCCCAAUGCCCTT |
| si-Importin β1 | GCUCAAACCACUAGUUAUATT | UAUAACUAGUGGUUUGAGCTT |
| si-Nup153 | GCUGCUGACACCAAAGUGATT | UCACUUUGGUGUCAGCAGCTT |
| si-Nup214 | GGCAGUGGGCAAACAGAAUTT | AUUCUGUUUGCCCACUGCCTT |
| si-Nup358 | GGAAGUCCUGCAAUUUAUATT | UAUAAAUUGCAGGACUUCCTT |
| si-NC | UUCUCCGAACGUGUCACG UTT | ACGUGACACGUUCGGAGAATT |
